# Edward John Kiepe, Ph.G., M.D.

**Published:** 1913-01

**Authors:** 


Edward John Kiepe, Ph.G., M.D., of Buffalo, died November 24th, 1912, aged 46. He entered the Buffalo College of Pharmacy as a student in 1889, graduating two years later with the degree of Ph.G. In 1894 he entered upon the study of medicine in the Medical Department of the University of Buffalo, receiving the doctor's degree in 1897. He was soon after called to the chair of Materia Medica in the department of Pharmacy and to that of Adjunct Professor of Materia Medica in the Department of Medicine, which positions he has filled continuously to the present time.

Professor Kiepe was much interested in the matter of scientific research and had prepared himself for such work by a post-graduate course in Johns Hopkins University and it was his expressed intention to continue his studies in Germany during the coming summer.

He was instrumental in organizing, in the University, the course of Pharmacology, now an important branch of scientific teaching in medical schools, and had done a considerable amount of work in animal experimentation in investigating the action of drugs in health and disease, aiding in elevating this branch of medical science to its present prominence. He was particularly interested in the most advanced ideas in therapeutics, in the application of which he had established a more than local reputation. He was the author of a text-book much used by students.

As an officer of the Pharmacy Faculty he served for many years as registrar and secretary, giving always of his best and bringing to every duty that painstaking ability, enthusiasm, faithfulness and devotion which characterized his every action in life. Of an intensely nervous temperment and more than usual sensitiveness, this close application to the affairs of the College together with a conscientious attention to the trying details of an exacting medical practice, united to induce a mental condition directly accountable for his untimely death.

Always a faithful friend, his generous nature and ready sympathy have endeared him to his associates and the thousands of students who have profited by his counsel and instruction. Those who were so fortunate as to have been associated with him intimately will regret the loss of his cheerful companionship, the ever-ready helpfulness and willingness with which, in true Christian spirit he assumed the burdens of others.

